# Duhamel versus transanal endorectal pull through (TERPT) for the surgical treatment of Hirschsprung’s disease

**DOI:** 10.1007/s10151-016-1524-5

**Published:** 2016-09-14

**Authors:** E. Arts, S. M. B. I. Botden, M. Lacher, P. Sloots, M. P. Stanton, I. Sugarman, T. Wester, I. de Blaauw

**Affiliations:** 1 Department of Surgery, Division of Pediatric Surgery, Radboudumc-Amalia Children’s Hospital, Nijmegen, The Netherlands; 2 Department of Pediatric Surgery, University of Leipzig, Leipzig, Germany; 3 Department of Pediatric Surgery, ErasmusMC-Sophia Children’s Hospital, Rotterdam, The Netherlands; 4 Department of Pediatric Surgery, University Hospital – Southampton General Hospital, Southampton, UK; 5 Department of Pediatric Surgery, Leeds Teaching Hospitals NHS Trust, Leeds, UK; 6 Department of Pediatric Surgery, Karolinska University Hospital, Solna, Stockholm Sweden

**Keywords:** Hirschsprung’s disease/surgery, Infant, Newborn, Proctocolectomy, Restorative/methods, Management of Hirschsprung’s disease

## Abstract

For the surgical treatment of Hirschsprung’s disease, several surgical techniques are used to resect the distal aganglionic colon. Two frequently used techniques are the Duhamel procedure and the transanal endorectal pull-through procedure. During the ‘8th Pediatric Colorectal Course’ in Nijmegen, November 2015, a workshop was organized to share experiences of both techniques by several experts in the field and to discuss (long term) outcomes. Specifically, the objective of the meeting was to discuss the main controversies in relation to the technical execution of both procedures in order to make an initial assessment of the limitations of available evidence for clinical decision-making and to formulate a set of preliminary recommendations for current clinical care and future research.

## Introduction

Hirschsprung’s disease (HD) is a congenital condition that is caused by the absence of ganglion cells in the submucosal and myenteric plexuses of the distal intestine [1, 2]. The surgical management of HD has moved from multi-stage open procedures to single-stage transanal surgical techniques [3]. Two frequently used techniques are the Duhamel retrorectal pull-through procedure [4, 5] and the transanal endorectal pull-through (TERPT) procedure [6]. Both techniques can involve laparoscopy as a means for taking biopsies to identify the transition zone and for mobilizing the colon [5]. In the Duhamel technique, a section of aganglionic rectum is left connected to a segment of ganglionic colon (side-to-side) as a pouch reservoir, whereas in the TERPT technique a very low direct anastomosis is made just above the dentate line [6, 7]. The latter can be done by leaving a muscular rectal cuff (Soave-like) or with a full-thickness resection of the distal colon and rectum (Swenson-like). More than two decades have passed since the implementation of the laparoscopic Duhamel and TERPT techniques as the treatment strategies for HD. However, there is an ongoing debate about many of the key issues, such as which technique is preferable and the execution and timing of these procedures. It is unclear if one of these techniques yields significantly better general and disease-specific outcomes.

The TERPT procedure, first developed by De La Torre and Langer, was a Soave-like transanal submucosal dissection with an endorectal pull-through, leaving an aganglionic rectal muscular cuff [6, 7]. This surgical procedure has also been modified to a transanal Swenson-like operation which does not require dissection in the submucosal plain but a straight resection of the full-thickness colon just above the dentate line [8]. Furthermore, a TERPT can be precluded by an open, transumbilical or laparoscopic biopsy for localization of the transition zone. Laparoscopy is then often used for mobilization of the aganglionic distal sigmoid colon.

When the Duhamel procedure is performed, the distal part of the aganglionic colon (rectum) remains in situ. After the resection, the ganglionic colon is placed in the avascular retrorectal plane and stapled or sutured side-to-side to the native aganglionic rectum [9, 10]. This was initially an open procedure; however, since the 1990s it has been performed laparoscopically with good results [9]. It can be a fully laparoscopic procedure, or a Pfannenstiel incision can be used when making the anastomosis [9, 10].

In recent years some centres of paediatric surgery have transitioned from performing the Duhamel procedure to performing the TERPT procedure in almost all cases [12, 13], but most surgeons appear to stick to their preferred surgical technique. There is little evidence supporting the superiority of procedure either in general or in specific cases [7, 8, 11–17], although some authors prefer the Duhamel procedure in long-segment disease. The available studies in which the short- and long-term outcomes of both techniques are investigated and compared often involve small heterogeneous patient samples and are frequently based on a retrospective or observational design, a combination which is likely to lead to biased results.

During the 8th Pediatric Colorectal Course and Workshops, in Nijmegen, the Netherlands, in November 2015, a ‘Surgical techniques & outcomes in Hirschsprung disease’ workshop was organized that focused on this topic. The aim of the meeting was to: (1) discuss the main controversies related to the technical execution of the TERPT and Duhamel procedures and (2) to formulate a set of preliminary recommendations for current clinical care and future research.

## Discussion

Experiences with both techniques differed greatly between the expert panel and participants in the workshop. It was agreed, however, by both the experts and the participants that the currently available evidence, particularly with regard to long-term outcomes, appears to be insufficient to significantly impact clinical decision. The consultants’ personal experience with a certain technique seems to have the strongest influence on the choice of treatment on each centre. In addition, there was a general consensus amongst experts that only highly skilled experienced surgeons could successfully perform the procedures, particularly the Duhamel procedure. It was agreed that a minimum of 20 cases was required to reach the plateau of the learning curve for each of the procedures, and generally training was not begun until fellow status was reached. Positive experiences with the Duhamel procedure that were shared by all experts were the limited anal stretching necessary for this procedure and the good visibility during the whole process. Also, the minimally invasive nature of the TERPT procedure and the good cosmesis were points of agreement.

In addition to these points of consensus, several controversial aspects of the surgical technique were discussed in the workshop; (1) the modus operandi for identifying the transitional zone by means of surgical biopsy, (2) the type of muscular cuff that is created in the TERPT technique and (3) the (native) pouch and/or spur left after removal of the aganglionic bowel using the Duhamel technique.

### Colonic biopsy

Before the actual process of removing the aganglionic bowel can be initiated, the transition zone between ganglionic bowel tissue and non-functioning aganglionic bowel needs to be determined. The gold standard for the diagnosis of HD is a biopsy [18, 19], for which several approaches are available; submucosal or seromuscular and transmural or full-thickness biopsies. Overall, (submucosal) rectal suction biopsy is initially performed for diagnostic purposes before the final surgery (TERPT or Duhamel). Submucosal biopsies are reported to be accurate, although reported sensitivity and specificity rates are 81–93 % and 97–98 %, respectively [20–22].

During surgery (open or laparoscopy), a seromuscular or a transmural full-thickness biopsy can be taken. A punch biopsy that samples seromuscular bowel tissue only is regarded as a simple, safe, fast and inexpensive method, although this type of biopsy may be more difficult for the pathologist to interpret [20, 23, 24]. In cases of partial circumferential aganglionosis, severe myenteric hypoganglionosis or hypertrophic submucosal nerves, a transmural full-thickness biopsy is more appropriate [25]. The risk of taking an individual section that is not through-and-through (full thickness) is that one may be sampling ganglionic tissue while underneath in the submucosa the bowel tissue is aganglionic or may display signs of neural dysplasia [26]. Leaving (partly) non-functional bowel (transitional zone pull-through) may lead to long-term complications such as constipation, obstructive symptoms with enterocolitis and widening of the distal colon [26].

The experts’ view on the preferred modus operandi for acquiring the biopsy during a TERPT or Duhamel procedure was the first point discussed during the workshop. The seromuscular biopsy was preferred by some experts since in their experience it had the advantage of a lower risk of spillage of bowel contents in the abdominal cavity and minimized the risk of perforation postoperatively. Several textbooks and published papers also mention seromuscular biopsies for diagnostics during laparoscopy [27], and the main advantage being that this method is minimally invasive and there is no spillage of bowel contents. The opinion of the experts was that the seromuscular biopsy may give more sampling errors if the transition zone is irregular.

A full-thickness biopsy on the other hand allows the pathologist to review all the layers of the bowel. Another method that can be used (particularly in open Duhamel procedures) is that of a circumferential doughnut biopsy taken intraoperatively. This allows the pathologist to review a sample in all quadrants rather than just the antimesenteric border, when a single-point biopsy is taken. This will certainly give the least errors. The potential benefit of a full-thickness biopsy or doughnut is that it will significantly decrease the number of patients with a transitional zone pull-through. Many of these patients require redo surgery although the exact numbers are not available [26]. The potential downside is a longer procedure every time a more proximal doughnut biopsy is required.

The experts also disagreed about the timing of the biopsy. TERPT is mostly initiated with a perineal dissection, and a biopsy is generally taken when a change in circumference or calibre of the colon is observed. However, several experts favoured a preliminary biopsy by means of laparoscopy or through an umbilical incision, before initiating the perineal dissection. The advantage of taking a preliminary biopsy is that it provides more certainty about the location of the transitional zone, which may be particularly important as this location may differ from the radiological findings [19, 28]. Consequently, the length of the aganglionic section of the colon may be underestimated, which could complicate a fully transanal approach [29]. In the light of this issue, another advantage of a (laparoscopically assisted) preliminary biopsy was discussed, namely that whilst waiting for the analysis of the frozen section by a pathologist (at least 30–40 min), the surgeon can proceed with the mobilization of the colon distal to the biopsy intra-abdominally. This may reduce the stress on the anal sphincters and also allows for a visual check for torsion of the bowel.

### Rectal muscular cuff versus no cuff

The original TERPT procedure described by De La Torre [6] and in a separate study by Langer [8] was a Soave-like procedure requiring a muscular rectal cuff to be dissected from the submucosa transanally. It can be technically challenging but was originally implemented to protect the surrounding tissue from surgical damage. More recently, a modification of the TERPT technique was described which involves going straight, full thickness, not mobilizing a rectal cuff by dissecting the submucosal plane (Swenson-like technique) [30, 31]. Several surgical key remarks were made on the subject. First of all, very often the procedures are started as a Soave-like procedure with a submucosal surgical plane in the first 0.5–1.0 cm and is then converted to full-thickness plane (Swenson-like technique), which does not require further submucosal dissection. However, it was observed that when using the Swenson plane, there is a risk that the surgeon inadvertently creates a plane too far from the rectum, gets lost and risks damaging structures anterior to the rectum (pelvic nerves, vagina, urethra/bladder neck).

Another point of discussion was the length of the cuff in the original De La Torre and Langer technique. The original descriptions of TERPT by Soave include a submucosal dissection above the peritoneal reflection to prevent injury to pelvic structures [6]. However, this approach, proposed by Soave, was not a transanal technique but was performed transabdominally. Pelvic floor structures were easily damaged if the surgeon did not operate flush on the rectal wall, and therefore, the idea of leaving the rectal outer wall as a cuff was born. In the transanal technique, the submucosal dissection starts just above the dentate line and originally ends at the peritoneal reflection [6, 7]. To prevent postoperative obstruction, it was reported by several experts that they would incise the rectal cuff, which was also described by Yang et al. [3, 32].

One of the major issues that were discussed by the participants was the length of the rectal cuff. A complete rectal cuff (from above the dentate line to the peritoneal reflection) may constrict the pull-through bowel because of its obvious aganglionic nature, even when it is incised at the end of the procedure. It has also been described in the literature that a long seromuscular cuff should be avoided as it can lead to obstruction, constipation, constriction and enterocolitis [33, 34] and this was the experts’ opinion as well. Determining the exact size or the ideal length of the cuff is not easy, but it was proposed that it should be no longer than at least 5 cm. This opinion is very subjective as in some studies the comparison of longer and shorter cuff lengths showed that the difference in associated postoperative complications was not statistically significant [33, 35]. Overall, at this meeting, the majority preferred using the Swenson approach for the TERPT procedure with a short cuff.

### Dysfunctional pouch

In the Duhamel procedure, a pouch is made with aganglionic tissue on the ventral side and ganglionic tissue on the dorsal side, whereas in the TERPT procedure almost all aganglionic tissues are removed and an end-to-end anastomosis is made 1 cm above the dentate line [9, 10]. It was discussed that the Duhamel technique may lead to specific complications, because of the native aganglionic rectal segment that remains in the new pouch after the reattachment to the healthy colon. As reported during the workshop, reattachment is performed by closing the top end of the native rectum and attaching the ganglionic bowel to the latero-posterior side of the aganglionic bowel making a distal pouch. However, it may leave enough tissue for a spur to be formed, which could impair proper bowel function. The preferred method was reported as stapling the opening of the pouch top to the wall of the pulled-through bowel, but incising the anterior wall of the pulled-down bowel and anastomosing this to the top of the pouch prevent pouch formation. The anterior wall of this pouch contains aganglionic tissue, which, if a spur is present, allows stool to sit there and lead to the anterior pouch expanding over time. A dilated pouch or a pouch that is left too long may lead to stasis of faeces, bacterial overgrowth, infection, obstruction and soiling [26, 36, 37]. In the experience of the attending experts, most of these mild symptoms generally improve. However, in more severe cases a redo operation is necessary, in which the Duhamel pouch is reduced in size [27, 37, 38] or has to be removed and converted to a TERPT [38]. Thus, the Duhamel pouch remains a reason for concern in a few cases for some of experts in the field.

### Long-term outcomes

Four long-term complications were considered essential by experts in patients with HD: obstructive symptoms (due to stricture, residual aganglionosis or transition zone, residual fibrosis or cuff tissue, torsion of distal colon), enterocolitis, incontinence and soiling. The experiences with these complications differed greatly amongst experts. The skill level of the surgeon was deemed of great importance for the success of the procedure and outcomes during long-term follow-up. There is only limited data available on long-term functional results after Duhamel or TERPT procedures. Systematic reviews by several groups report on research that explores outcomes of the Duhamel and TERPT techniques and predominantly include small, observational studies with limited follow-up length and heterogeneous patient samples [13, 39, 40]. Limitations in the quality of study design make it difficult to interpret results [40]. Also, in the opinion of the experts, the definitions of outcome parameters such as surgical complications, enterocolitis, constipation and incontinence differ greatly in the literature are not standardized for (longitudinal) monitoring of the outcome. There was a general consensus that the majority of HD patients experience problems with bowel function during follow-up, regardless of the technique that is used. Most experts said they find it difficult to properly monitor bowel function objectively during long-term follow-up as the child ages due to lack of a suitable measurement tool. There are many different types of outcome measures available such as the Rintala, Holschneider and Kelly scoring tools [41] or the enterocolitis definition proposed by Pastor et al. [42]. However, not all of these measurement tools are properly validated in the HD population and there does not appear to be one single scoring system that is generally accepted [41]. There are many well-validated bowel function scoring systems for adults, which may be of some use in assessing outcomes of adolescents in terms of soiling, for example, although it would be essential for paediatric surgeons to agree which is most appropriate.

Bias in the reporting of outcomes may exist, since in the studies it is often operating surgeon who monitors long-term outcome [41]. Interestingly, it was discussed that despite the possible source of bias, this set-up also has advantages as it guarantees continuity of care and benefits the patient–doctor relationship. Most participants strongly preferred that set-up. On the other hand, there was a general consensus about the need for an objective, standardized HD-specific scoring system for the monitoring of long-term outcomes during follow-up that preferably also touches on quality of life related to general health or gastrointestinal symptoms [43]. This may require someone not directly involved in the treatment to assist with follow-up assessment and the development and validation of a HD-specific instrument for long-term monitoring [41].

## Conclusions

Although the evolution of surgical techniques in the treatment of HD has provided tremendous improvement in patient outcomes, there are certain aspects of treatment that could be improved. As regards the Duhamel versus TERPT technique, evidence is insufficient to recommend one technique over the other and the surgeon’s experience is the key factor determining the choice of procedure. Perhaps these techniques could be applied more efficiently in certain subgroups, which have to be determined. This expert workshop led to the recommendation for a systematic review on the technical execution of the Duhamel and TERPT procedures.

Summary of the main consensus points and recommendations:

- The experience of the main surgeon is likely to have a major effect on long-term outcomes. As there currently is no evidence to suggest one technique has significant superiority over another, the technique, the surgeon is most experienced with, is recommended.
- Duhamel or TERPT may be the best for specific subgroups of patients, but this needs further research and evidence. The TERPT technique may be preferable in straightforward cases, with a more distal localization of aganglionosis, and the Duhamel technique is favourable when treating patients with long-segment disease affecting the proximal colon.
- Laparoscopy is preferred over open techniques to assist in a Duhamel and/or transanal pull-through procedure.
- A preliminary biopsy (preferably full thickness) and laparoscopic intraabdominal mobilization of the proximal colon should be considered to reduce stress on the anal sphincter (TERPT) and permit proper localization of the transitional zone.
- There is a need for objective and clear definitions of both the surgical techniques and the primary long-term outcomes (bowel function) preferably including quality of life.
